# Novel compound heterozygous mutations in *TELO2* in an infant with You-Hoover-Fong syndrome: A case report and literature review

**DOI:** 10.1515/biol-2022-0602

**Published:** 2023-05-19

**Authors:** Yong Zhao, Yu Han, Nuo Li, Wenjie Fu, Guanjun Luo, Yuan Tan, Xuguang Qian

**Affiliations:** Department of Children Rehabilitation, Nanhai Women and Children’s Hospital Affiliated to Guangzhou University of Traditional Chinese Medicine, No. 6, Guiping West Road, Nanhai District, Foshan, Guangdong Province, 528200, China

**Keywords:** You-Hoover-Fong syndrome, *TELO2*, compound heterozygous mutation, congenital cataract, developmental retardation

## Abstract

We report here the clinical diagnosis and treatment and genetic mutations of an infant with You-Hoover-Fong syndrome (YHFS). The relevant literature review was conducted. A female infant aged 17 months was admitted to Nanhai Affiliated Maternity and Children’s Hospital of Guangzhou University of Chinese Medicine due to “global development delay complicated with postnatal growth retardation for more than 1 year.” The infant was diagnosed with YHFS due to the onset of extremely severe mental retardation, microcephaly, abnormal hearing, severe protein–energy malnutrition, congenital cataract, cleft palate (I°), congenital atrial septal defect, brain atrophy, hydrocephalus, and brain hypoplasia. The whole exon sequencing revealed two compound heterozygous mutations, including a likely pathogenic *TELO2* variant, c.2245A > T (p.K749X) from her mother and an uncertain variant, c.2299C > T (p.R767C) from her father, validated by Sanger sequencing. After bilateral cataract surgery, the infant obtained better visual acuity and showed more responses and interactions with her parents. Diagnosis and treatment of this case prompt that these *TELO2* variants have not been reported, deepening the understanding of the molecular and genetic mechanism of YHFS in clinical practice.

## 1 Introduction

You-Hoover-Fong syndrome (YHFS) (OMIM: 616954), also known as TELO2-related syndrome, is a severe syndrome caused by the loss of double allele of telomere maintenance 2 (*TELO2*) of 16p13, which is mostly autosomal recessive.

In yeast, *TEL2* is involved in telomere length regulation [1]. However, unlike yeast, *TELO2* does not participate in telomere maintenance in humans but plays a vital role in cell stress response [2]. The study in 2016 [3] found that *TELO2* mutation affected the stability of the triple T complex (TTT; *TELO*2, Tel two-interacting protein 1 (*TTI1*), and Tel two-interacting protein 2 (*TTI2*)) but did not affect the length of telomeres. The specific pathogenic mechanism remains to be further elucidated.

YHFS is extremely rare, and since its first description in 2016, only ten individuals have been reported, all cases showing a severe disability [4]. The main manifestations include microcephaly, hearing impairment, ataxia, global developmental retardation, aphasia, short finger/oblique finger/toe syndactyly, abnormal sleep pattern, seizures, and nystagmus [5].

*TELO2* forms a part of the co-chaperone TTT complex, along with *TTI1* (OMIM: 614425) and *TTI2* (OMIM: 614426). This complex collectively plays a critical role in the maturation and stabilization of the phosphatidylinositol 3-kinase-related protein kinases (PIKKs).

In recent years, significant advancement has been achieved in the identification of potential mutational hotspots by *in silico* mutagenesis. Ankita and Rituraj [6] have successfully enhanced the acid resistance of serratiopeptidase via implementing computational interventions to screen out the most stable mutational hotspot. In addition, Vidya [7] reported a comprehensive atomic behavior of the gain-of-function mutation (R132H) in the IDH1 enzyme, which provides a direction toward new therapeutics. Vidya et al. [8] organized a long-term molecular dynamics simulation (500 ns) and performed comparative conformational analysis, demonstrating that due to mutation at 315th position (threonine to isoleucine), original structures deviated from normal and attained a flexible conformation.

Recent studies have demonstrated that compound heterozygous loss-of-function mutations in *TELO2* are associated with the incidence and progression of YHFS [9]. Here we reported a female infant diagnosed with YHFS, accompanied by extremely severe mental retardation, microcephaly, abnormal hearing, severe protein-energy malnutrition, congenital cataract, cleft palate (I°), congenital atrial septal defect, brain atrophy, hydrocephalus, and brain hypoplasia. The literature review was also carried out to identify novel *TELO2* mutations in YHFS and analyze the clinical features, aiming to deepen the understanding of this extremely rare disease in clinical practice.

## 2 Case presentation

### 2.1 Baseline data

The female child, aged 17 months, was admitted to our hospital on December 9th, 2020, because of delayed physical and intellectual development complicated by slow growth and development. At the age of 1 month, her limbs were tense and unresponsive, and she received family rehabilitation in a local hospital, and at the age of 10 months, she received systematic rehabilitation treatment, whereas low efficacy was obtained. On December 9th, 2020, she was transferred to our hospital. She was able to raise her head, roll over, and sit for several minutes but failed to climb or stand up. She could grasp things with bilateral hands but could not pinch. After birth, the child was breastfed and supplemented with formula milk powder at 2 months. She had poor appetite, chewing, and swallowing function. At 3 months, she could raise her head, roll over at 10 months, and sit independently at the age of 1 year. At 4 months, she could laugh, laugh aloud at 8 months, and recognize her parents when 1 year old. The infant was delivered at a gestational age of 35 + 6 weeks, weighed 1.49 kg, had a head circumference of 29 cm, and a height of 41 cm. The 1 min Apgar score was 9, 9 at 5 min, and 10 at 10 min. She was hospitalized due to extremely low birth weight, premature infant, congenital cataract, neonatal hyperglycemia, cleft palate I°, persistent superior vena cava, patent foramen ovale, patent ductus arteriosus, mild tricuspid regurgitation, and pulmonary hypertension. Maternal prenatal examination showed that fetal development was slow, and persistent superior vena cava and lateral ventricle were widened. She underwent “extracapsular cataract extraction + anterior vitrectomy + posterior capsulotomy” in a local hospital on May 8th, 2020 (7 months old) and September 16th, 2020 (11 months old), respectively.


**Informed consent:** Informed consent has been obtained from all individuals included in this study.
**Ethical approval:** The research related to human use has been complied with all the relevant national regulations, institutional policies, and in accordance with the tenets of the Helsinki Declaration, and has been approved by the authors’ institutional review board or equivalent committee.

## 3 Physical examination

**Figure 1:**
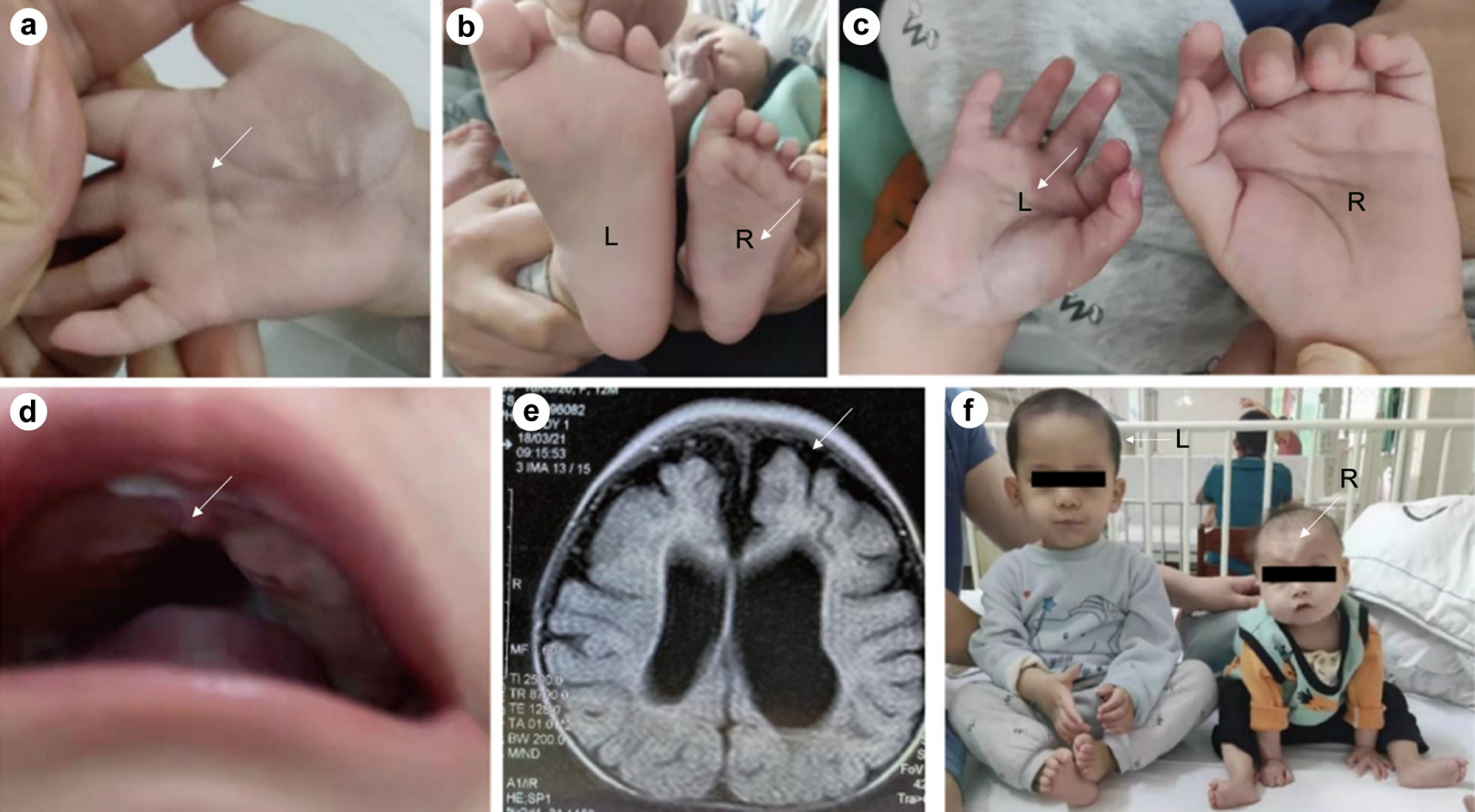
Physical features and MRI of the brain of the girl. (a) Simian crease was found in the right palm; (b) comparison of the foot size between the patient (R) and healthy counterpart (L); (c) comparison of the palm size between the patient (L) and healthy counterpart (R); (d) I° cleft palate; (e) MRI findings of hydrocephalus; (f) comparison of physical characteristics between the patient (R) and healthy counterpart (L).

Upon admission, the child was conscious and in normal mental status, malnourished, emaciated (weighed 4.7 kg), body height of 63 cm, pale skin, simian crease was found in the right palm, small-size limbs (Figure 1a–c), small head circumference (36.5 cm), anterior fontanelle closure, normal eye movement, occasional tremor, and no strabismus. Bilateral pupils were round and equal in size with light reflection. The tongue was normal. A 1 cm long cleft was seen in the upper jaw (Figure 1d). No abnormal pulsation was detected in the precordial area. Heart rate was 120 beats/min with normal rhythm and no pathological murmur. She was able to raise head and roll over, sit for several minutes but could not climb or stand up. She could grasp things with bilateral hands but could not pinch. Grade 1 muscle tension of four limbs (MAS classification), grade 3 muscle strength of four limbs, moderate joint mobility, knee tendon reflex and Achilles tendon reflex were active, and ankle clonus and Tripod sign were negative, while Babinski sign was positive.

## 4 Clinical diagnosis and treatment

### 4.1 Laboratory and imaging examinations

Routine blood, urine, and stool tests yielded normal results. No abnormality in liver and kidney function was found. Bone alkaline phosphatase was 200 U/L, hepatitis B and Treponema pallidum-specific antibody were negative, while cytomegalovirus IgG antibody was positive. ECG yielded generally normal results. Color Doppler echocardiography prompted central-type congenital heart disease, normal left ventricular systolic and diastolic function, and CDFI showed horizontal left-to-right shunt of the atrium. Spiral PT plain scan of the ear: bilateral horizontal semicircular canal dysplasia and poor gasification of bilateral mastoid and femoral cavity were considered, BAEP: abnormal results for bilateral ears, genetic screening for hereditary deafness detected no gene mutation. Fundus examination found a mature retina and retinal pigmentation. Eyeball ultrasound hinted at the diagnosis of congenital cataract, FERG: the wave amplitude of binocular FERG dark and light adaptation was lower than the normal range, bilateral F-VEP: the latency of P2 wave was not delayed, and the amplitude was significantly reduced (preoperative); F-VEP: the latency of F-VEP P2 wave in the right eye was not delayed, but the amplitude was slightly decreased. The latency of the F-VEP P2 wave in the left eye was not delayed, whereas the amplitude was slightly decreased (postoperative). Cranial MRI prompted the diagnosis of corpus callosum dysplasia, brain atrophy, and supratentorial communicating hydrocephalus (Figure 1e). EEG: Abnormal findings with slow background rhythm.

### 4.2 Behavioral assessment by different tools

Gesell Developmental Schedules consists of five domains, including social adaption, gross motor, fine motor, language, and social development. The child obtained 15.1 in the domain of social adaption, 17.3 in gross motor, 18.8 in fine motor, 24.1 in language, and 16.0 in social development.

Gross motor score of SM scale = 0, lower than the normal value of 8. Mental development index of Bayley Scales of Infant and Toddler Development (3rd edition) <50, psychomotor development index <50. Peabody Developmental Motor Scales (2nd edition): gross motor score = 10, equivalent to the value of a 2-month-old infant, below 1st percentile; vision-motor coordination score = 3, equivalent to the value of a 2-month-old infant, below 1st percentile; fine motor score ≤2, developmental quotient ≤46, below 1st percentile.

### 4.3 Genetic testing

**Figure 2:**
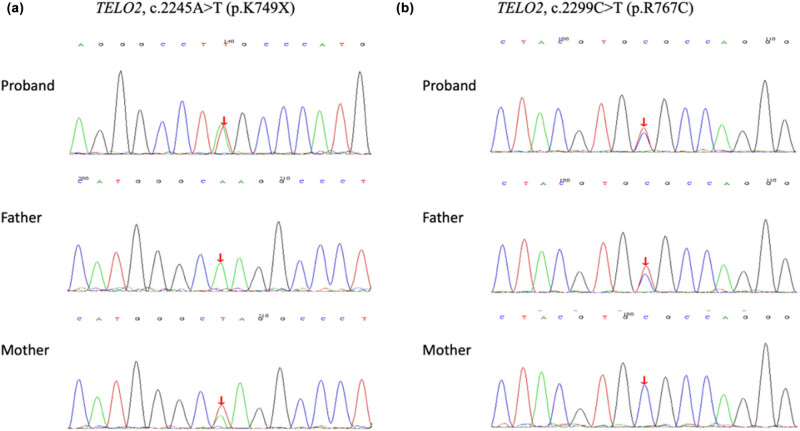
Sanger sequencing reveals *TELO2* mutations in the affected girl and her parents. (a) A likely pathogenic *TELO2* variant, c.2245A > T (p.K749X), from her mother; (b) an uncertain variant, c.2299C > T (p.R767C), from her father.

Genetic metabolic disease detection revealed no abnormality. SMA detected no defective gene. Chromosome aberration test detected no chromosome-induced genome copy number variation, no changes in the number and structure; Mitochondrial genome: no phenotype-related definite pathogenic variation was found; Karyotype and microarray: no definite pathogenic variation was detected; Blood tandem mass spectrometry, a test to identify 48 genetic diseases associated with metabolisms of amino acids, organic acids, and fatty acid oxidation, by analyzing tens of metabolites in the blood (–). Whole exome sequencing (WES): 23,000 genes (49.11 Mb) were detected, and 11,101 loci of 3,086 genes related to the phenotype of the patient were obtained. Subsequently, the WES results were further validated by Sanger sequencing, identifying two *TELO2* mutations in the affected girl and her parents. A likely pathogenic *TELO2* variant, c.2245A > T (p.K749X), was detected from her mother (Figure 2a), and an uncertain variant, c.2299C > T (p.R767C), was identified from her father (Figure 2b).

## 5 Rehabilitation therapy

Upon initial admission, the infant was able to roll over but unable to support herself using her hands. Additionally, she was unresponsive, poor in spirit, had poor limb movement, and had trouble with chewing, swallowing, and pronunciation. After admission, physical therapy and massage were performed to promote the development of gross motor movement. Head acupuncture was given in the motor, foot sensory, visual, and language areas. Eye, ear, and tongue acupuncture were applied to promote motor development and improve visual acuity, hearing, and swallowing capability. Physical therapy has been assigned to improve brain function and promote exercise and intellectual development. Intravenous drip was delivered to improve cerebral circulation and nourish cranial nerves. Moxibustion was prescribed to strengthen physical fitness and promote growth and development. Vitamin D and calcium were orally given to prevent rickets. Oral drugs were given to supplement the substrates essential for nerve development. After one course of treatment, the infant could sit independently for several minutes, became more sensitive and responsive, and showed more interaction with her parents.

## 6 Discussion

In 2016, You et al. [3] first reported six affected individuals from four families with intellectual disability and neurological and other congenital abnormalities associated with compound heterozygous variants in *TELO2*, which is subsequently described as YHFS, a syndromic intellectual disability disorder caused by variants in *TELO2* (MIM: 611140), which is the human ortholog of Tel2, an *S. cerevisiae* gene identified in a screen for genes involved in the maintenance of telomere length [1]. Located at 16p13, *TELO2* has 21 exons and encodes an 837 amino acid protein that interacts physically with TELO2 interacting proteins 1 and 2 (TTI1 and TTI2) to form the TTT complex consisting of *TELO2*, *TTI1*, and *TTI2*. TTT complex acts as a co-chaperone for the maturation of the PIKKs, which participate in the processes of DNA breakage and replication and play a significant role in human growth and development [1].

You et al. [3] performed WES of the affected individuals and their parents in family 1 and identified rare compound heterozygous missense variants in TELO2: c.1100G > T (p.Cys367Phe) in exon 8 and c.2159A > T (p.Asp720Val) in exon 18. The proband in family 2 is heterozygous for c.1100G > T (p.Cys367Phe), and a rare heterozygous missense variant c.2296G > A (p.Val766Met). The proband in family 3 is a compound heterozygote for c.779C > T (p.Pro260Leu) and c.1826G > A (p.Arg609His). The proband in family 4 is a compound heterozygote for p.Asp720Val, and a complex allele of c.514C > T (p.Gln172X) plus c.2034 + 1G > A (IVS16 + 1G > A), respectively. Interestingly, the characterization of patient fibroblasts revealed reduced protein levels of TELO2 and other components of the TTT complex, but no significant changes were observed for downstream PIKK functions [3]. More specifically, telomere length in the patients was comparable to those in the controls.

In 2017, Moosa et al. [9] reported two Danish sisters with a severe expression of YHFS and identified the frameshift variant in *TELO2*, located in exon 14, c.1750dupA, which is predicted to lead to premature termination of the protein, p.Thr584Asnfs*42. The second *TELO2* variant, c.2312T > C, is located in exon 20. While in 2020, Del-Prado-Sánchez et al. [5] reported two siblings diagnosed with YHFS at the age of 28 and 14 months. Both were genetically studied to find the cause of their developmental delay and microcephaly. The identical compound heterozygous missense mutations in the *TELO2* gene were found in each sibling. In 2021, Ciaccio et al. [4] described the case of two sisters who presented a milder phenotype of *TELO2*-related syndrome due to carrying the homozygous p. Arg609His variant of *TELO2*. They reported that such variant had been reported once in a more severely affected patient, in the compound heterozygous state associated with p. Pro260Leu variant, suggesting a potential role of p. Arg609His variant in determining milder phenotypes. More severe cases were presented with severely impaired motor and language skills [9], which were also observed in our case. It was proposed that truncating variants, such as c.1750dupA (p.Thr584Asnfs*42), correlate with more severe clinical manifestations, whereas most missense variants, such as p.Arg609His are associated with milder symptoms due to their more limited functional consequences [4,9]. Our patient also carried one truncating variant c.2245A > T (p.K749X), which may contribute to the overall severe phenotypes. Further functional study on this nonsense variant and the other missense variant (c.2299C > T, p.R767C) will help clarify their individual contributions to the disease outcome.

In one most recent report of *TELO2*-related disease, 14 new individuals were added to the 12 individuals mentioned above [10].

Taken together, the 17-month-old female infant presents with a typical expression of YHFS, carrying two compound heterozygous mutations, including a likely pathogenic *TELO2* variant, c.2245A > T (p.K749X), from her mother and an uncertain variant, c.2299C > T (p.R767C), from her father. These two *TELO2* variants have not been reported, which expands the spectrums of genotypes and phenotypes for this extremely rare disease of *TELO2*-related YHFS. Nevertheless, this is merely a single case report. These preliminary findings remain to be validated by subsequent clinical studies with a larger sample size.
